# The Association Between Uterine Artery Pulsatility Index at Mid-Gestation and the Method of Conception: A Cohort Study

**DOI:** 10.3390/medicina61061093

**Published:** 2025-06-16

**Authors:** Antonios Siargkas, Ioannis Tsakiridis, Dimitra Kappou, Apostolos Mamopoulos, Ioannis Papastefanou, Themistoklis Dagklis

**Affiliations:** 1 Third Department of Obstetrics and Gynecology, School of Medicine, Faculty of Health Sciences, Aristotle University of Thessaloniki, 54124 Thessaloniki, Greeceamamop@auth.gr (A.M.); 2 Institute of Life, IASO General Hospital, 15123 Athens, Greece; d.kappou@yahoo.com; 3 Fetal Medicine Clinic, Monis Petraki 4, 11521 Athens, Greece; 4 Department of Women and Children’s Health, Faculty of Life Sciences and Medicine, King’s College London, London WC2R 2LS, UK

**Keywords:** uterine artery pulsatility index, assisted reproductive technology, in vitro fertilization, intracytoplasmic sperm injection, ovulation induction, intrauterine insemination, second trimester doppler, placental function, uteroplacental perfusion, mid-trimester ultrasound

## Abstract

*Background and Objectives*: Pregnancies resulting from assisted reproductive technology (ART) have been associated with placenta-related adverse outcomes. Uterine artery Doppler pulsatility index (UtA-PI) reflects placental function. This study aimed to examine whether second-trimester UtA-PI differs according to the conception method after adjusting for potential confounding factors. *Materials and Methods*: In this retrospective cohort study, we included data from February 2015 to August 2024, at the third Department of Obstetrics and Gynecology, School of Medicine, Faculty of Health Sciences, Aristotle University of Thessaloniki, Greece, on singleton pregnancies presenting for their routine antenatal care, including a second-trimester anomaly scan. Pregnancies conceived via ART, including those conceived via ovulation induction/intrauterine insemination (OI/IUI) or in vitro fertilization/intracytoplasmic sperm injection (IVF/ICSI), were compared to those conceived spontaneously. Multiple linear regression was employed to investigate the association between the mode of conception and log_10_ UtA-PI values, adjusting for various confounders, including gestational age at the time of the scan, maternal weight, height, age, parity, mode of delivery, smoking status, pre-existing diabetes mellitus (type I or II), and pre-existing thyroid disease. *Results*: The study included 15,552 singleton pregnancies, of which 82 (0.5%) were conceived via OI/IUI and 690 (4.4%) were conceived via IVF/ICSI. The median UtA-PI values were 0.99 (IQR: 0.85–1.17) for spontaneous conception (SC), 1.00 (IQR: 0.86–1.16) for OI/IUI, and 0.90 (IQR: 0.76–1.12) for IVF/ICSI. The Kruskal–Wallis test indicated a statistically significant difference among these groups (*p* < 0.001). Pairwise comparisons using the Wilcoxon rank-sum test with Bonferroni correction revealed that UtA-PI values in IVF/ICSI pregnancies were significantly lower compared to both SC and OI/IUI pregnancies (*p* < 0.001 for both). No significant difference was observed between the SC and OI/IUI groups. In the multivariable linear regression model, IVF/ICSI conception was independently associated with lower log_10_ UtA-PI values (estimate = −0.076, 95% CI: −0.096, −0.056) while no association was found for OI/IUI conception. *Conclusions*: Although ART has been associated with placental-related complications, mid-trimester UtA flow was found to be lower in IVF/ICSI pregnancies, suggesting better utero-placental flow in ART pregnancies and other possible mechanisms in the maternal–placental interplay for the development of pregnancy complications.

## 1. Introduction

Uterine artery pulsatility index (UtA-PI), measured via Doppler ultrasound during pregnancy, quantifies the impedance to blood flow within the uterine arteries supplying the placenta [1,2]. UtA-PI reflects placental function and has been associated with fetal growth and maternal cardiovascular condition [3]. Preeclampsia (PE), fetal growth restriction (FGR), and small-for-gestational-age (SGA) neonates are linked with placental dysfunction and may result in maternal and neonatal morbidity and mortality [4,5,6].

PE is a leading contributor to maternal and perinatal morbidity and mortality, affecting approximately 2–3% of pregnancies [7]. In recent years, efforts have focused on its early identification and prevention. UtA-PI is a key component in first-trimester combined screening algorithms for PE, guiding the prophylactic administration of low-dose aspirin to women identified as high-risk and reducing the incidence of preterm PE, early placental dysfunction, and severe preterm birth, as recent data suggest [8,9,10]. Additionally, in the second trimester, UtA-PI is also integrated into multivariate prognostic models as an established marker for placenta-mediated complications, including PE, SGA, FGR, and stillbirth [11,12]. Importantly, the inclusion of UtA-PI in mid-trimester risk prediction models not only enhances the early identification of high-risk pregnancies but also supports the implementation of timely clinical interventions. These interventions may contribute to improved perinatal outcomes and potentially reduce the economic burden on healthcare systems [11,12,13,14,15].

In healthy, low-risk pregnancies, UtA-PI steadily declines with advancing gestational age, reflecting the normal reduction in utero-placental resistance as pregnancy progresses, while it is also affected by various maternal and pregnancy-specific parameters [16]. Physiologic data also show that the expected late-gestation fall in UtA-PI is coupled with a rise in total uterine-artery blood flow, an adaptation that supports placental perfusion and is positively associated with higher neonatal birth weight [17]. Advanced maternal age is associated with a lower UtA-PI, and similarly, higher maternal body mass index (BMI) correlates with a decreased UtA-PI; these effects are especially evident in early pregnancy [16]. Parity has a lasting influence as nulliparous women tend to have a higher UtA-PI compared to multiparous women, consistent with the permanent uterine vascular changes that follow the first birth [18]. Another recent study suggests that parous women with preeclampsia in a previous pregnancy have higher UtA-PIs in the index pregnancy [19]. In contrast, maternal smoking is linked to an increase in UtA-PI, indicating higher uterine arterial resistance [20].

Over the past four decades, assisted reproductive technology (ART) has transitioned from an experimental approach into a widespread fertility treatment, resulting in over 10 million births through in vitro fertilization (IVF) worldwide [21]. ART is considered a risk factor for placental dysfunction-related conditions [22,23,24]. Therefore, important clinical considerations emerge about the applicability of UtA-PI, a known marker of placental dysfunction, in ART pregnancies. Limited evidence exists on the levels of UtA-PI values in ART and spontaneously conceived pregnancies (SC), especially in the second trimester. Most existing studies have evaluated UtA-PI during the first trimester, with some reporting lower UtA-PI values in IVF pregnancies compared to SC [25,26], while others found no significant differences [27,28]. To date, only one study has directly evaluated UtA-PI during mid-gestation, suggesting reduced values in IVF pregnancies, though it did not adjust for potential confounders [25]. Another investigation that included mid-trimester data focused solely on pregnancies achieved through oocyte donation and similarly reported lower UtA-PI values compared to SC [29]. Given the physiological and clinical relevance of the second trimester in assessing uteroplacental perfusion and the scarcity of robust data, further research is warranted to clarify how the mode of conception influences UtA-PI during this crucial window of gestation.

Our central hypothesis was that pregnancies conceived via ART would exhibit significantly different UtA-PI values compared to SC. This study, therefore, aimed to examine this hypothesized difference and to assess its persistence after adjustment for potential confounding factors in a large cohort of unselected singleton pregnancies.

## 2. Methods

### 2.1. Study Design and Setting

This retrospective cohort study utilized data collected from February 2015 to August 2024 at the third Department of Obstetrics and Gynecology, School of Medicine, Faculty of Health Sciences, Aristotle University of Thessaloniki, Greece. All participants received routine antenatal care at the above-mentioned institution.

### 2.2. Ethical Considerations and Reporting Standards

Ethical approval for this study was granted by the Research Ethics Committee of the School of Medicine, Aristotle University of Thessaloniki, on 20 September 2024 (Reference: 320/2024). Prior to inclusion, all participants gave informed consent for the anonymized use of their clinical data for research purposes. The reporting of this observational study adheres to the principles outlined in the Strengthening the Reporting of Observational Studies in Epidemiology (STROBE) guidelines [30]. Furthermore, the development and reporting of the analytical model followed the Transparent Reporting of a Multivariable Prediction Model for Individual Prognosis or Diagnosis (TRIPOD) recommendations [31].

### 2.3. Study Population and Data Collection

Eligible participants included all consecutive singleton pregnancies receiving antenatal care at our institution, confirmed viable from 20^+0^ weeks until 23^+6^ weeks of gestation that had also attended for the first trimester a nuchal scan in the same department. Exclusion criteria were multiple gestations, singleton pregnancies with known fetal genetic or structural anomalies, termination of pregnancy, miscarriages before 23^+6^ weeks, and incomplete follow-up data. Clinical data, including maternal demographics, anthropometrics, obstetric history, lifestyle factors, method of conception, mode of delivery, and pre-existing medical conditions, were systematically collected following a standardized protocol and recorded in an electronic database (Astraia).

### 2.4. Assisted Reproductive Technology Groups

The study group consisted of women who conceived via ART, as defined by the International Committee for Monitoring Assisted Reproductive Technology (ICMART), and the control group consisted of SC [32]. For comparative purposes, ART pregnancies were further classified into two groups, ovulation induction (OI) or intrauterine insemination (IUI) and IVF or intracytoplasmic sperm injection (ICSI). We combined OI with IUI and IVF with ICSI because each technique on its own had too few pregnancies to give reliable results; pooling them into less-invasive (OI/IUI) and more-invasive (IVF/ICSI) groups boosted the numbers enough to make a meaningful comparison. Both fresh and frozen embryos were used in the IVF/ICSI group.

### 2.5. Fetal Medicine Assessment

Standardized second-trimester ultrasound examinations were performed by five sonographers certified by the Fetal Medicine Foundation (London, UK), who had extensive training in ultrasound scanning and had obtained the appropriate Fetal Medicine Foundation Certificate of Competence in ultrasound and Doppler examinations, ensuring consistency in the examination protocol, image quality, and measurement techniques. For these measurements, a 4–9 MHz transducer was used (Voluson E8, GE Healthcare, Austria, Zipf). The ultrasound scan included examination for fetal anatomy and measurement of fetal head circumference, abdominal circumference, and femur length to calculate the EFW. The UtA Doppler was measured transabdominally following the precise steps outlined in the ISUOG guidelines for the second trimester [33].

For transabdominal UtA-PI measurement in the second trimester, the probe is placed longitudinally in the lower lateral abdomen and angled medially in the parasagittal plane. Color flow mapping helps identify the uterine artery as it crosses the external iliac artery. The artery typically runs along the uterus toward the fundus, and the probe should be adjusted to align with its course. The sample volume is placed 1 cm distal to the crossover point. If the artery bifurcates before this point, the sample is taken just before the split. The same steps are repeated on the opposite side. As pregnancy advances, dextrorotation of the uterus causes the left artery to appear less lateral than the right [33].

UtA-PI was calculated by measuring both the left and right UtA-PI, and their average was used for statistical analysis. The calculation of UtA-PI followed this formula: UtA-PI = (UtA peak systolic velocity − UtA end-diastolic velocity)/time-averaged maximum velocity. Gestational age was determined from measurement of fetal crown–rump length at 11–13 weeks [34].

### 2.6. Statistical Analysis

Descriptive statistics were initially used to summarize participant characteristics, with continuous variables presented as the mean ± standard deviation (SD) or median ± interquartile range (IQR), depending on their normality distribution. Categorical variables were reported as counts and percentages. Differences in these baseline characteristics among the three conception method groups were evaluated using one-way ANOVA for normally distributed continuous variables, Kruskal–Wallis tests for non-normally distributed continuous variables, and chi-squared tests for categorical variables. We then generated boxplots to visually compare the distribution of UtA-PIs among the conception groups. Due to the observed right-skewness of the UtA-PI measurements, a base-10 logarithmic transformation (log_10_ UtA-PI) was applied to approximate a normal distribution, facilitating its use in regression modeling (Figures S1 and S2).

To assess the independent relationship between the mode of conception and uterine artery Doppler indices, a multiple linear regression model was constructed with log_10_ UtA-PI as the dependent variable. The predictors were conception method categories and a series of potential confounding factors such as gestational age (weeks) at the time of the scan, maternal weight (kilograms), height (centimeters), age (years), parity, smoking status, history of previous cesarean section, pre-existing diabetes mellitus (type I or II), and pre-existing thyroid disease. A final, parsimonious model was derived through backward elimination from the initial forced entry model on the basis of statistical significance. The assumptions of our multivariable linear regression were assessed using the residuals versus fitted values plot (Figure S3) and the normal Q-Q plot of residuals (Figure S4), while multi-collinearity was evaluated with the Variance Inflation Factor (VIF). The results of the regression analyses are presented as parameter estimates along with their corresponding 95% confidence intervals (CIs). All statistical procedures were performed using R software, version 4.4.2 (R Foundation for Statistical Computing, Vienna, Austria).

## 3. Results

### 3.1. Cohort

**Figure 1:**
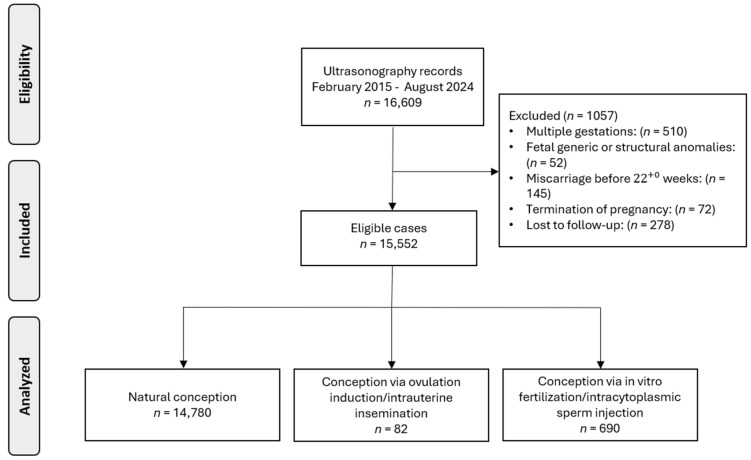
Flowchart of the population selection process.

Our study initially included data from 16,609 pregnant women. Following the exclusion of cases with multiple gestations (*n* = 510), fetal genetic or structural anomalies (*n* = 52), miscarriage before 22 + 0 weeks (*n* = 145), termination of pregnancy (*n* = 72) and incomplete follow-up data (*n* = 278), our final analysis encompassed 15,552 singleton pregnancies (Figure 1). This cohort consisted of 82 (0.5%) pregnancies conceived via OI/IUI and 690 (4.4%) conceived through IVF/ICSI.

### 3.2. Baseline Characteristics

Women conceiving via OI/IUI or IVF/ICSI were significantly older, taller, and more frequently nulliparous compared to those who conceived spontaneously. Additionally, women who conceived via IVF/ICSI exhibited a higher prevalence of pre-existing diabetes mellitus, thyroid disease, and gestational diabetes mellitus, while their neonates had decreased birth weight compared to the SC. Women using ART were less likely to smoke compared to SC. Table 1 summarizes the characteristics of these groups.

### 3.3. Distribution of Uterine Artery Doppler Pulsatility Index

**Figure 2:**
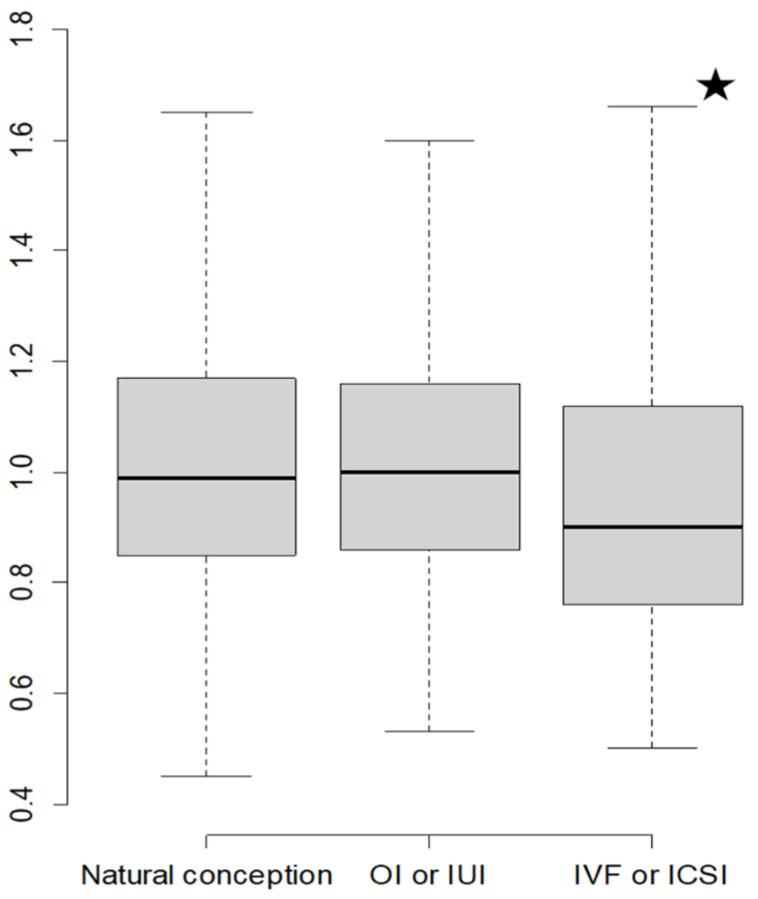
Boxplots depicting the mean uterine artery pulsatility index relative to the method of conception. Abbreviations: ICSI, intracytoplasmic sperm injection; IUI, intrauterine insemination; IVF, in vitro fertilization; OI, ovulation induction. The star symbol in the figure denotes statistical significance compared to the rest of the groups with the Wilcoxon rank sum test with Bonferroni correction.

**Figure 3:**
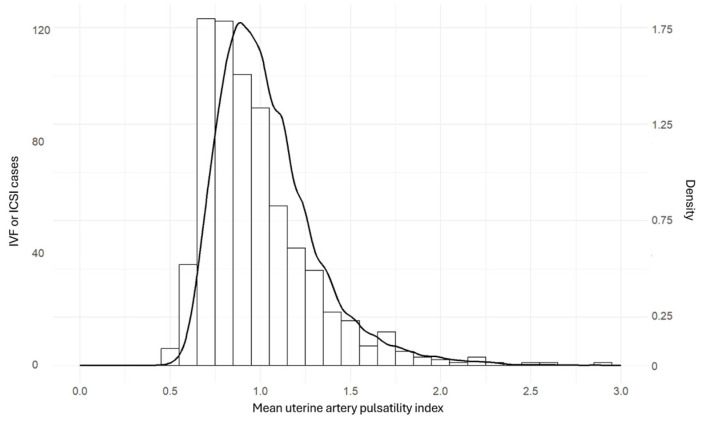
Histogram depicting the distribution of the mean uterine artery pulsatility index (UtA-PI) in pregnancies conceived via in vitro fertilization or intracytoplasmic sperm injection conception. Abbreviations: ICSI, intracytoplasmic sperm injection; IVF, in vitro fertilization. The superimposed line demonstrates the UtA-PI in pregnancies resulting from spontaneous conception.

Median UtA-PI values were 0.99 (IQR: 0.85–1.17) for SC, 1.00 (IQR: 0.86–1.16) for OI/IUI, and 0.90 (IQR: 0.76–1.12) for IVF/ICSI. The Kruskal–Wallis test indicated a statistically significant difference among these groups (*p* < 0.001). Pairwise comparisons using the Wilcoxon rank sum test with Bonferroni correction showed significant differences between IVF/ICSI UtA-PI and both SC and OI/IUI pregnancies (*p* < 0.001). The boxplot and the histogram illustrate these differences (Figure 2 and Figure 3).

### 3.4. Multivariate Modelling

**Figure 4:**
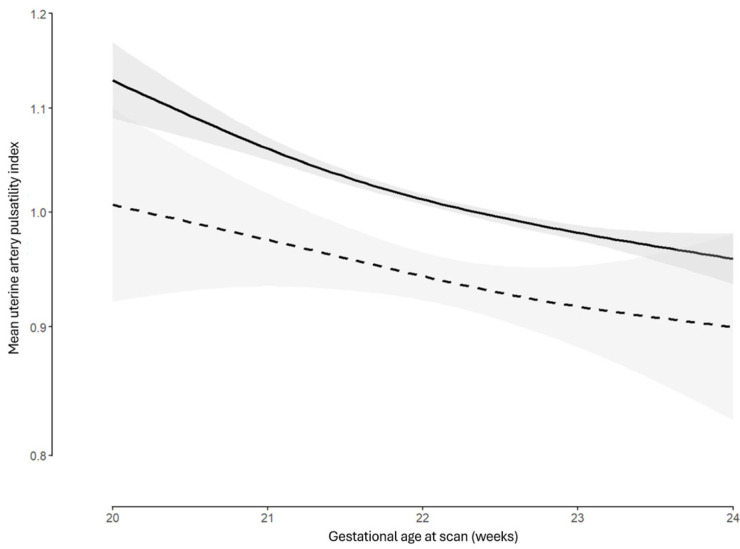
Uterine artery pulsatility index between 20 + 0 and 23 + 6 weeks in natural conception versus in vitro fertilization or intracytoplasmic sperm injection conception. Solid line, natural conception; dashed line, in vitro fertilization or intracytoplasmic sperm injection conception; gray area, 95% confidence intervals.

Multivariable linear regression analysis evaluated the independent association between conception methods and log_10_ UtA-PI, adjusting for relevant confounders. The final regression model demonstrated a significant negative association between log_10_ UtA-PI and IVF/ICSI conception (Table 2). In contrast, OI/IUI was not associated with log_10_ UtA-PI (Table 2). Log_10_ UtA-PI values were positively associated with maternal age and parity, specifically previous vaginal delivery, and inversely related to maternal weight and height. Log_10_ UtA-PI shows a curvilinear reduction with advancing gestational age (Figure 4). The curve for IVF/ICSI pregnancies consistently lies below that of SC, indicating lower UtA-PI values across the 20^+0^–23^+6^ gestational week window (Figure 4). Regression diagnostics, specifically, the residuals versus fitted values plot (Figure S3), the normal Q-Q plot of residuals (Figure S4), and the calculated VIF, revealed that the linear regression assumptions were not violated (Figures S3 and S4).

## 4. Discussion

### 4.1. Main Findings

This large cohort study demonstrates that mid-trimester UtA-PI is significantly lower in pregnancies conceived via IVF/ICSI compared to those conceived spontaneously (Figure 2 and Figure 3). This association remained statistically significant after adjusting for multiple confounding variables (Table 2). Conversely, pregnancies conceived via OI/IUI exhibited no significant difference in UtA-PI compared to SC.

### 4.2. Comparison with Previous Studies

Our analysis indicates that, following adjustments, IVF/ICSI pregnancies exhibit lower UtA-PI values compared to SC (Figure 2, Figure 3 and Figure 4). The existing body of research on this specific topic is limited, and to our knowledge, this is the first large study to account for confounding factors (Table 2). A relevant prospective cohort study involving 1962 pregnancies observed consistently lower UtA-PI values throughout gestation in IVF pregnancies compared to SC, including second trimester measurements [25]. While that study established new reference ranges for the UtA-PI in IVF pregnancies that were consistently lower than those of SC, it acknowledged the limitations posed by unadjusted differences between the two groups [25].

Another small study, focusing exclusively on pregnancies from oocyte donations versus SC, included 296 women. It measured UtA-PI in every trimester, including mid-gestation, and after adjusting for gestational age, parity, and weight, this study reported a significant coefficient estimate of −0.37 for log_10_ UtA-PI in the oocyte donation group [29]. A prospective first-trimester screening study of 27,461 pregnancies found a reduced UtA-PI in IVF pregnancies in univariate analysis, but this association lost statistical significance after adjustments [35]. In that study, the ovulation induction group showed a slight, non-significant increase in UtA-PI [35].

### 4.3. The Role of Confounders

The role of confounders is pivotal in the study of UtA-PI to derive clinically meaningful results, as highlighted in both the existing literature [16,18,20] and our current research. Examining the influence of confounding factors across diverse populations is also crucial for ensuring the broad applicability of research findings. This is particularly important in IVF pregnancies, which frequently present with a higher burden of baseline risk factors (Table 1), as inadequate adjustment can lead to spurious associations and misinterpretations of IVF’s effect on pregnancy vascular adaptation. Overall, our results concerning UtA-PI confounders align with previous research. Consistent with Tayyar et al. [16], we observed a decrease in UtA-PI with advancing pregnancy and no association with maternal age in the second trimester. Additionally, maternal height and weight demonstrated a positive association with UtA-PI, corroborating previous reports [36]. Our analysis of parous women differentiated between those with prior cesarean sections and those with only vaginal deliveries. We observed a novel positive association between a history of only vaginal deliveries and UtA-PI. While the existing literature generally indicates lower UtA-PI in nulliparous women, these associations may be less pronounced in the second trimester, as suggested by Tayyar et al. [16]. Our review of the literature revealed only one study comparing women with prior vaginal delivery to nulliparous women, which did not demonstrate a statistically significant association in the second trimester [37].

### 4.4. Interpretation of Findings

The underlying reasons and mechanisms for the lower UtA-PI in IVF/ICSI pregnancies remain unclear, with limited research available. A retrospective study of 27,289 pregnancies provided some insight, examining UtA-PI in the first trimester [26]. This study found no significant difference in UtA-PI for the OI/IUI group or fresh embryo transfer within the IVF group, compared to SC. However, both natural cycle and artificial cycle frozen embryo transfers in the IVF group showed a lower UtA-PI than SC, with the artificial cycle frozen embryo transfer group demonstrating a statistically significant difference [26]. This pattern is further supported by a review suggesting that frozen embryo transfers may exhibit better uterine perfusion, resulting in higher birthweight and lower risk of SGA neonates compared to fresh embryo transfers [38]. This suggests that the lower UtA-PI observed in the IVF group might be primarily associated with artificial cycle frozen embryo transfers. A potential explanation is that the lower UtA-PI seen in pregnancies following artificial cycle frozen embryo transfers compared to natural cycle frozen embryo transfers could be related to a hyperestrogenic state induced by exogenous estrogen administration up to the 10th week of gestation [39]. Inversetti et al. also observed a lower UtA-PI in ICSI with egg donation conceptions compared to SC at 11^+0^ to 13^+6^ weeks [40]. Further supporting the potential role of hormonal treatments, a retrospective cross-sectional study found that pregnancies resulting from hormone replacement therapy had lower UtA-PI compared to natural cycle pregnancies [41]. The authors hypothesized that the lower resistance in the hormone replacement therapy group might result from the administration of high-dose vaginal progesterone, given that progesterone is known to exert a vasodilatory effect, leading to decreased uterine artery impedance [39].

### 4.5. Clinical Implications

Lower mid-trimester UtA-PI in IVF/ICSI pregnancies is paradoxical given their well-documented excess risk of placental disease [20,21,22]. A recent meta-analysis confirmed a substantially elevated PE risk associated with IVF/ICSI (overall ~50% increase), with risk varying by specific protocol (fresh transfer: +50%; frozen transfer: +80%; oocyte donation: +500%) compared to SC [42]. Factors like advanced maternal age, comorbidities, and underlying infertility diagnoses (e.g., ovulatory disorders) contribute to a high risk profile in ART pregnancies. However, a prominent hypothesis implicates delayed trophoblastic invasion and impaired placentation, potentially manifesting as an increased UtA-PI, as a key mediator [43]. During normal pregnancy, spiral arteries undergo significant remodeling to become low-resistance vessels, allowing for increased blood flow to the placenta. This process involves changes in the decidual and myometrial segments of the spiral arteries, driven by both decidual and trophoblast-associated stages. In cases of impaired placentation, this remodeling process is incomplete, resulting in higher resistance to blood flow in the uterine arteries. This increased resistance is commonly reflected in a higher UtA-PI, indicating reduced uteroplacental perfusion [44]. However, relevant studies that have reported reduced UtA-PIs for various ART methods, such as IVF/ICSI, particularly when involving egg donation and artificial cycle, have detected increased risk for adverse outcomes such as PE, SGA, and preterm delivery for these groups [26,28,29,45,46]. This is a significant finding as it may show that among IVF/ICSI pregnancies, the UtA-PI may be lower even when there is placental dysfunction and not as trustworthy as it is among SC.

Our findings further support that the increased risk of placental disease in IVF/ICSI may be driven by impaired placentation, which may not be detectable via mid-trimester UtA-PI. This raises a potential clinical concern: a low UtA-PI measurement in an IVF/ICSI pregnancy, interpreted against standard reference ranges derived from SC, could provide false reassurance despite the inherently higher baseline risk of placental dysfunction-related disorders in ART pregnancies. Another important clinical implication underscored by our findings is the clinical necessity for a combined multivariate assessment in IVF pregnancies to, first, adjust for various factors affecting UtA-PI levels and, second, to take into account other important risk factors present in IVF pregnancies to overcome the fact that UtA-PI levels may not perform well in identifying IVF pregnancies at risk for placental related disorders.

### 4.6. Strengths and Limitations

A notable strength of this study is its large sample size and the systematic approach to data collection. All sonographers involved were certified by the Fetal Medicine Foundation, ensuring a high-quality assessment with standardized ultrasound measurements. Additionally, the use of multivariate analysis fosters safe conclusions by adjusting for the influence of confounders. The prospectively collected data from an unselected population under a pre-specified antenatal protocol is a major strength that enhances the validity of the clinical interpretation.

However, certain limitations should be acknowledged. The first is the retrospective nature of the study, although it does not appear to introduce any bias in our inferences. Second, while we adjusted for many confounders, residual confounding, particularly from specific ART protocols or lifestyle factors, cannot be entirely excluded. Third, the single-center design might limit the generalizability of the findings to other populations or healthcare settings. Fourth, we did not include multiple pregnancies; therefore, the findings cannot be extrapolated to these cases. Fifth, we used pooled study groups, so we do not know how each ART method affects UtA-PI individually. Finally, the study establishes an association but does not elucidate the underlying biological mechanisms for the observed differences in UtA-PI.

### 4.7. Future Research and Perspectives

Drawing from our findings, future research should focus on elucidating the underlying mechanisms contributing to the observed lower UtA-PI in IVF/ICSI pregnancies despite the established higher risk of placenta-related complications. Longitudinal studies assessing UtA-PI throughout gestation in both ART and SC, with detailed stratification by specific ART methods, protocols (e.g., frozen vs. fresh embryo transfer, stimulation protocols), and medications used, would provide a more comprehensive understanding of the evolution of utero-placental function. Furthermore, integrating other markers of placental function, such as biochemical markers like placental growth factor, could offer a more holistic view of the maternal–placental interplay and its impact on pregnancy outcomes [44]. It is also crucial to investigate how this decrease in UtA-PI levels evident in IVF pregnancies is associated with the occurrence of adverse pregnancy outcomes in IVF/ICSI pregnancies. Specifically, future studies should examine whether more subtle variations in UtA-PI within the IVF/ICSI group correlate with a heightened risk of specific complications, compared to the risk associated with similar UtA-PI changes in SC and also investigate the longer-term effects [47,48].

## 5. Conclusions

This study has demonstrated that singleton pregnancies following IVF/ICSI exhibit significantly lower mid-trimester UtA-PIs and possibly improved utero-placental flow, compared to SC. Although ART has been associated with placenta-related complications, mid-trimester uterine artery flow appears to be better in IVF/ICSI-conceived pregnancies, suggesting other possible mechanisms in the maternal–placental interplay for the development of pregnancy complications. In the clinical front, the current UtA-PI reference ranges, derived from SC, may not be applicable to IVF/ICSI pregnancies. This disparity should be considered when aiming to achieve an accurate risk assessment and clinical management. Further research is imperative to focus on the underlying pathophysiology and the potential clinical implications of these differences, in the context of placental dysfunction following ART.

## Figures and Tables

**Table 1:** Characteristics of the examined cohort.

	Spontaneous Conception	OI/IUI	IVF/ICSI	*p*-Value
N	14,780	82	690	
Maternal age mean (SD) *	31.03 (5.14)	33.58 (4.78)	37.20 (5.37)	<0.001
Maternal weight, median [IQR] †	64.00 [57.00, 73.15]	63.00 [57.12, 72.00]	64.00 [58.00, 73.00]	0.239
Height, median [IQR] †	165.00 [160.00, 170.00]	167.00 [162.00, 170.00]	165.00 [162.00, 170.00]	0.015
BMI, median [IQR] †	23.10 [20.80, 26.60]	23.00 [20.90, 25.40]	23.00 [21.10, 26.70]	0.727
Nulliparous, *n* (%) ‡	7380 (49.9)	68 (82.9)	561 (81.3)	<0.001
Previous vaginal delivery, *n* (%) ‡	4389 (29.7)	8 (9.8)	43 (6.2)	
Previous cesarean section, *n* (%) ‡	3011 (20.4)	6 (7.3)	86 (12.5)	
Pre-existing diabetes mellitus (I or II), *n* (%) ‡	59 (0.4)	0 (0.0)	9 (1.3)	0.002
Thyroid disease, *n* (%) ‡	1068 (7.2)	8 (9.8)	96 (13.9)	<0.001
Smoker, *n* (%) ‡	2018 (13.7)	3 (3.7)	49 (7.1)	<0.001
Gestational diabetes mellitus, *n* (%) ‡	1285 (8.7)	8 (9.8)	108 (15.7)	<0.001
Gestational age in weeks, median [IQR] †	22.14 [21.71, 22.57]	22.00 [21.71, 22.29]	22.14 [21.71, 22.43]	0.023
No early diastolic notching, *n* (%) ‡	13,910 (94.1)	74 (90.2)	657 (95.2)	0.517
Unilateral early diastolic notching, *n* (%) ‡	646 (4.4)	5 (6.1)	25 (3.6)	
Bilateral early diastolic notching, *n* (%) ‡	216 (1.5)	3 (3.7)	8 (1.2)	
Birthweight in grams, mean (SD) *	3232.35 (500.10)	3075.41 (581.97)	3094.09 (588.70)	<0.001

Abbreviations: ICSI, intracytoplasmic sperm injection; IQR, interquartile range; IUI, Intrauterine insemination; IVF, in vitro fertilization; OI, ovulation induction; UtA PI, uterine artery pulsatility index; NA, not available; SD, standard deviation; *, normal continuous variables expressed as the mean and standard deviation were compared with one-way analysis of variance; †, non-normal continuous variable expressed as median and interquartile range were compared with the Kruskal–Wallis test; ‡, categorical variable expressed as percentages were compared with the Chi-square test.

**Table 2:** Multivariable linear regression models exploring the association between log_10_ uterine artery pulsatility index and conception via assisted reproduction technologies.

	Forced Entry Initial Model		Final Model	
Variables	Estimate (95% CI)	*p*-Value	Estimate (95% CI)	*p*-Value
Intercept	6.183 (3.303, 9.062)	<0.001	6.194 (3.315, 9.073)	<0.001
Method of conception				
Spontaneous conception	(Reference)		(Reference)	
OI or IUI	0.035 (−0.020, 0.090)	0.213	0.035 (−0.020, 0.090)	0.214
IVF or ICSI	−0.076 (−0.096, −0.056)	<0.001	−0.076 (−0.096, −0.056)	<0.001
Gestational age	−0.492 (−0.750, −0.233)	<0.001	−0.493 (−0.751, −0.234)	<0.001
Gestational age^2^	0.010 (0.004, 0.016)	0.001	0.010 (0.004, 0.016)	0.001
Weight	−0.002 (−0.004, −0.000)	0.018	−0.002 (−0.004, −0.000)	0.017
Weight^2^	0.000 (0.000, 0.000)	0.007	0.000 (0.000, 0.000)	0.007
Height	−0.002 (−0.002, −0.001)	<0.001	−0.002 (−0.002, −0.001)	<0.001
Maternal age	0.001 (0.000, 0.002)	0.007	0.001 (0.000, 0.002)	0.006
Parity				
Nulliparous	(Reference)		(Reference)	
Previous vaginal delivery	0.017 (0.007, 0.027)	0.001	0.017 (0.007, 0.027)	0.001
Previous cesarean section	−0.003 (−0.014, 0.008)	0.610	−0.003 (−0.014, 0.008)	0.600
Smoking	0.003 (−0.009, 0.015)	0.577	-	-
Pre-existing diabetes mellitus (I or II)	−0.021 (−0.081, 0.039)	0.492	-	-
Thyroid disease	0.006 (−0.009, 0.020)	0.464	-	-
Standard deviation	0.246		0.246	
R^2^	0.017		0.017	
AIC	516.7		511	

Abbreviations: AIC, Akaike information criterion; CI, confidence intervals; ICSI, intracytoplasmic sperm injection; IUI, intrauterine insemination; IVF, in vitro fertilization; OI, ovulation induction.

## Data Availability

The data presented in this study are available from the corresponding author upon reasonable request. The data are not publicly available due to privacy restrictions.

## Supplementary Materials

The following supporting information can be downloaded at https://www.mdpi.com/article/10.3390/medicina61061093/s1. Figure S1: Distribution of mean uterine artery pulsatility index in our population; Figure S2: Distribution of the Log_10_ of mean uterine artery pulsatility index in our population; Figure S3: Diagnostic Plot: Residuals versus fitted values for the final multiple linear regression model; Figure S4: Diagnostic Plot: Normal Q-Q plot of residuals for the final multiple linear regression model.
