# Supplementary material for: The Association Between Uterine Artery Pulsatility Index at Mid-Gestation and the Method of Conception: A Cohort Study
Source: Medicina (Kaunas). 2025 Jun 16;61(6):1093. doi: 10.3390/medicina61061093 (PMC12195363; doi:10.3390/medicina61061093)

Supplementary Figure S1. Distribution of mean uterine artery pulsatility index in our population.

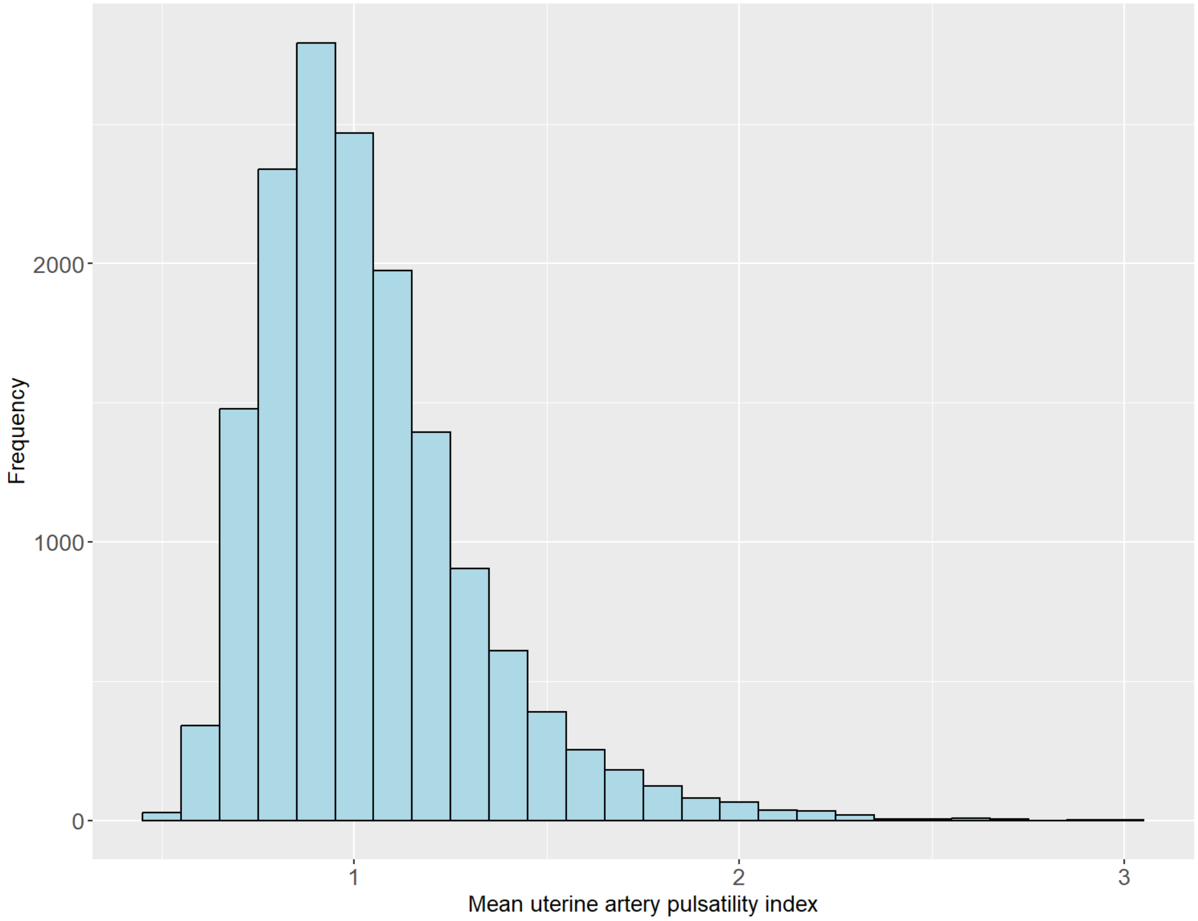

Supplementary Figure S2. Distribution of the Log_{10} of mean uterine artery pulsatility index in our population.

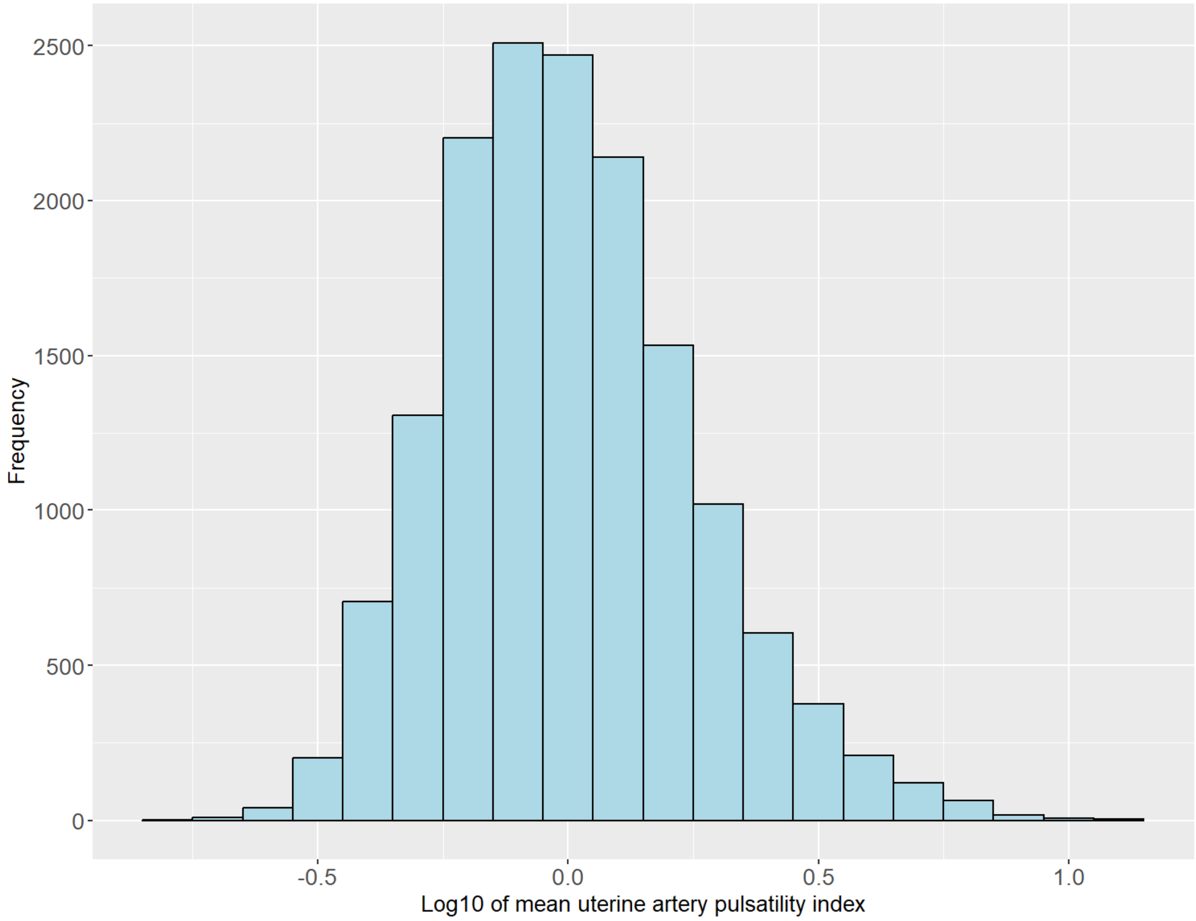

Supplementary Figure S3. Diagnostic Plot: Residuals versus fitted values for the final multiple linear regression model

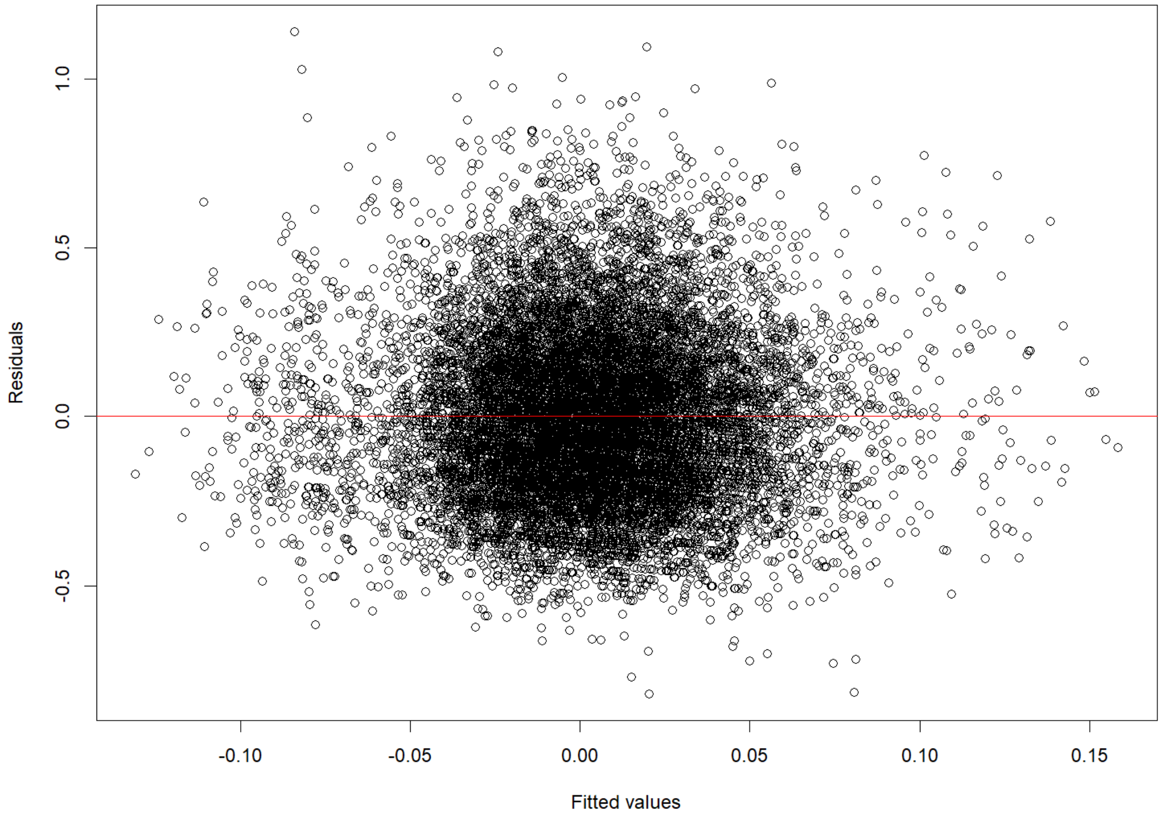

Supplementary Figure S4. Diagnostic Plot: Normal Q-Q plot of residuals for the final multiple linear regression model

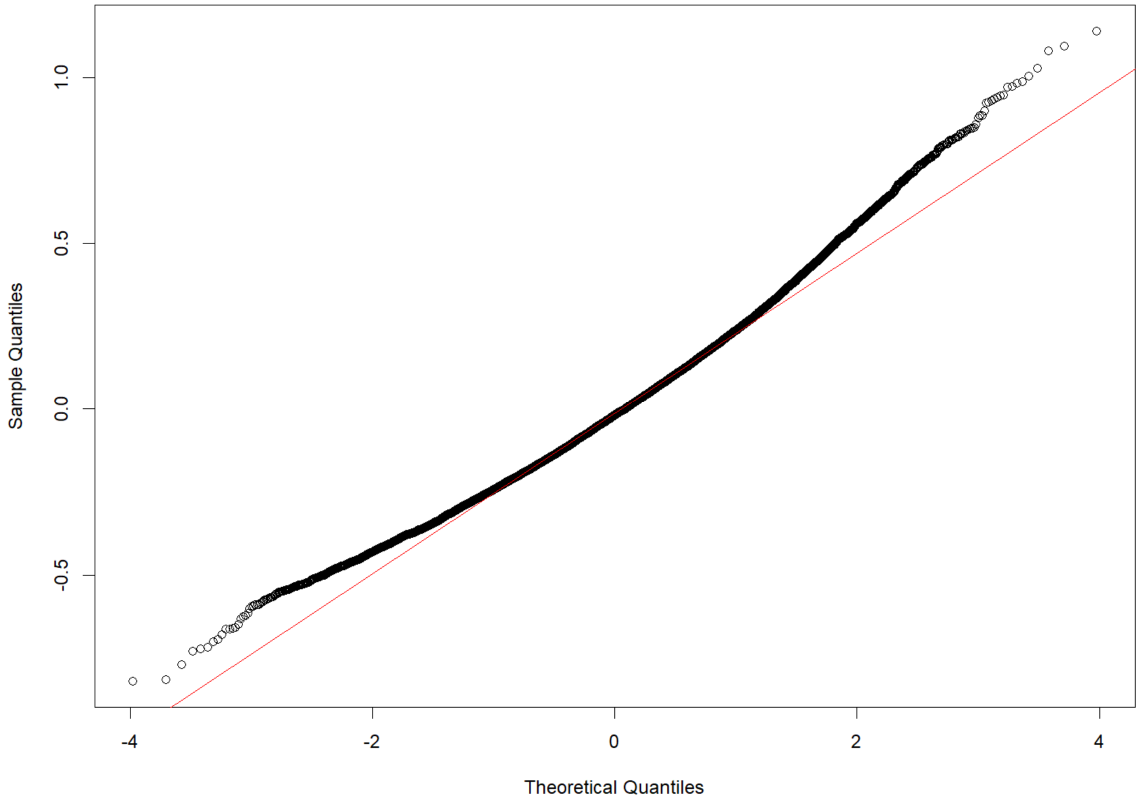

Supplement: Supplementary file 1 [file medicina-61-01093-s001.zip › medicina-3665500-supplementary.pdf]
